# Protein Profiles for Muscle Development and Intramuscular Fat Accumulation at Different Post-Hatching Ages in Chickens

**DOI:** 10.1371/journal.pone.0159722

**Published:** 2016-08-10

**Authors:** Jie Liu, Ruiqi Fu, Ranran Liu, Guiping Zhao, Maiqing Zheng, Huanxian Cui, Qinghe Li, Jiao Song, Jie Wang, Jie Wen

**Affiliations:** Institute of Animal Sciences, Chinese Academy of Agricultural Sciences, Beijing 100193, P. R. China; Universitat de Lleida, SPAIN

## Abstract

Muscle development and growth influences the efficiency of poultry meat production, and is closely related to deposition of intramuscular fat (IMF), which is crucial in meat quality. To clarify the molecular mechanisms underlying muscle development and IMF deposition in chickens, protein expression profiles were examined in the breast muscle of Beijing-You chickens at ages 1, 56, 98 and 140 days, using isobaric tags for relative and absolute quantification (iTRAQ). Two hundred and four of 494 proteins were expressed differentially. The expression profile at day 1 differed greatly from those at day 56, 98 and 140. KEGG pathway analysis of differential protein expression from pair-wise comparisons (day 1 vs. 56; 56 vs. 98; 98 vs. 140), showed that the fatty acid degradation pathway was more active during the stage from day 1 to 56 than at other periods. This was consistent with the change in IMF content, which was highest at day 1 and declined dramatically thereafter. When muscle growth was most rapid (days 56–98), pathways involved in muscle development were dominant, including hypertrophic cardiomyopathy, dilated cardiomyopathy, cardiac muscle contraction, tight junctions and focal adhesion. In contrast with hatchlings, the fatty acid degradation pathway was downregulated from day 98 to 140, which was consistent with the period for IMF deposition following rapid muscle growth. Changes in some key specific proteins, including fast skeletal muscle troponin T isoform, aldehyde dehydrogenase 1A1 and apolipoprotein A1, were verified by Western blotting, and could be potential biomarkers for IMF deposition in chickens. Protein–protein interaction networks showed that ribosome-related functional modules were clustered in all three stages. However, the functional module involved in the metabolic pathway was only clustered in the first stage (day 1 vs. 56). This study improves our understanding of the molecular mechanisms underlying muscle development and IMF deposition in chickens.

## Introduction

Improvement in lifestyle and changes in consumption habits mean that livestock production aims to provide sufficient meat of improved quality. Meat quality and production are influenced by intramuscular fat (IMF) content and skeletal muscle development. For example, the content of IMF and the myofiber type can affect meat quality traits such as flavor, juiciness, water holding capacity and tenderness[1–4]. In chickens, IMF is not visible and not anatomically separable, which makes it difficult to investigate the mechanism of its deposition. Protein profiles of whole muscle, therefore, are important in understanding the mechanisms for both muscle development and IMF deposition.

The short lifespan of chickens makes them an excellent model for studying various aspects of development. Some of the molecular markers of muscle structure/metabolism in livestock have also been identified by genome scans, but no studies using proteomics technology have linked muscle growth and IMF content in chickens. It is desirable to analyze the expression profile of proteins in chicken skeletal muscle at different ages[5,6]. Many studies have characterized proteins from 2-DE gels in pigs [7], cattle[8,9], and layers [5] at different stages of embryonic development to early growth after hatching. Doherty et al[5] have characterized the proteome of layer chicken breast muscle using two-dimensional gel electrophoresis (2-DE) from 1 to 27 days after hatching. Fifty-one proteins had mass spectra that matched existing chicken proteins in on-line databases. For many of these proteins, there were dramatic changes in relative expression levels during the 27 days of growth. Proteomic profiling of the breast muscle of Thai indigenous chickens during the growth period were also analyzed using the 2-DE method. A total of 259, 161, 120 and 107 protein spots were found to be expressed in the chicken breast muscles at 0, 3, 6 and 18 weeks of age, respectively. From these proteins, five distinct spots were significantly associated with chicken age. These were characterized and showed homology with phosphoglycerate mutase 1 (PGAM1), apolipoprotein A1 (APOA1), triosephosphate isomerase 1 (TPI1), heat shock protein 25 kDa (HSP25) and fatty acid binding protein 3 (FABP3)[10]. In addition, by application of an isobaric tagging for relative and absolute quantification (iTRAQ)-based approach, the proteomes of bovine embryos at the zygote and 2-cell and 4-cell stage with MII oocytes as a reference were quantitatively analyzed[11]. Bioinformatic analysis of 87 proteins that differed significantly in abundance between the four stages revealed proteins involved in the p53 pathway, lipid metabolism, and mitosis, indicating that these processes may play pivotal roles in embryonic development[11]. All of these studies showed the utility of proteomics as a tool for uncovering the molecular basis of physiological differences in muscle during these growth periods. Compared with the methods previously used (1D and 2D gels), the isobaric tags for relative and absolute quantitation (iTRAQ) analysis in the present study is more accurate and has been widely applied to investigate the proteome of different organisms at different developmental stages [12–15].

The present study used advanced proteomics methodology (iTRAQ) to identify differentially expressed proteins in breast muscles of slow-growing chickens at different post-hatching ages.

## Materials and Methods

### Ethics Statement

All of the animal experiments were conducted in accordance with the Guidelines for Experimental Animals established by the Ministry of Science and Technology (Beijing, China). Animal experiments were approved by the Science Research Department (in charge of animal welfare issue) of the Institute of Animal Sciences, CAAS (Beijing, China).

### Animals

Forty female Beijing-You chickens were obtained at day 1 from the Institute of Animal Science, Chinese Academy of Agricultural Sciences (Beijing, China), and were randomly assigned to four groups of 10. Individuals were reared in stairstep caging under continuous lighting using standard conditions of temperature, humidity and ventilation. The same diet was fed to all chickens and a three-phase feeding system was used: the starter ration (d 1 to d 28) with 21.0% crude protein and 12.12 MJ/kg, the second phase (d 28 to d 56) with 19.0% crude protein and 12.54 MJ/kg, and the last phase (after d 56) with 16.0% crude protein and 12.96 MJ/kg. Feed and water were provided ad libitum during the experiment. All birds were fasted for 12 h, and weighed before being killed by stunning and exsanguination. The left breast muscles were collected from 10 chickens at day 1 (hatching), 56 (fast growth age), 98 (marketing age) and 140 (first egg age). All samples (200–300 mg) were snap-frozen and stored at −80°C before analysis. The entire right breast and livers were collected, weighed and stored at −20°C for phenotypic measurement.

### IMF measurement

IMF content of breast and fat content of the liver were determined by Soxhlet extraction, as described previously[16,17], and expressed as percentages of the dry weight of the breast muscle.

### Protein extraction

Frozen breast muscle tissues (~200 mg) was homogenized in 1 mL lysis buffer containing 7 M urea (Sigma, St Louis, MO, USA), 2 M thiourea (Sigma), 4% (w/v) 3–3 (cholamidopropyl) dimethylammonio-1-propanesulfonate (CHAPS; Sigma), 65 mM dithiothreitol (DTT; Sigma), and 0.05% (w/v) protease inhibitor (Sigma). The homogenates were held on ice for 30 min and centrifuged for 30 min at 12,000 *g*, to remove insoluble components. The total protein concentration of each sample was determined with a 2-D Quant kit (GE Healthcare, Pittsburgh, PA, USA). The ten samples of each age group were pooled using equal amounts of protein then the four pools were diluted to the same concentration with Tris-buffered saline (TBS) before iTRAQ labeling. The samples were stored at −80°C until analysis. Each pool was tested twice.

### iTRAQ labeling

After precipitation with acetone, the protein (200 μg) of each pool was dissolved with 1 M DTT for 1 h at 37°C and kept in the dark with 1 M indole-3-acetic acid (IAA) for 1 h at room temperature. Samples were dissolved and centrifuged twice with 120 μl UA (8 M urea in 0.1 M Tris.HCl, pH 8.5), and then re-dissolved and centrifuged three times with 100 M lautyltrethylammonium bromide (LTEAB) (1 M). The proteins (2–4 μg) were digested with trypsin (trypsin: protein = 1:50; Sigma) and incubated at 37°C overnight. Each peptide pool was then passed through a 0.2-μm centrifugal filter for 20 min at 10,000 *g* at 20°C. Labeling of each pooled sample was 2-plex, where two reporter tags were used; hatchling samples were labeled with reporter tags 113 and 117; those from day 56 with reporter tags 114 and 118; the pool at day 98 with reporter tags 115 and 119; and the last pool from day 140 was labeled with reporter tags 116 and 121. The four 2-plex labeled samples were then combined into a single 8-plex sample mixture and dried by centrifugal evaporation.

### Strong cation exchange (SCX) separation and reverse phase liquid chromatography tandem mass spectrometry (RPLC-MS/MS)

The combined peptide mixture was analyzed by RPLC-MS/MS for simultaneous identification and quantification. The sequence of a peptide is determined from the products that are generated from proteolytic cleavage of the protein and the relative quantity of a given peptide among the treated samples is determined from the intensities of reporter ion signals also present in the MS/MS scan. iTRAQ-8 plex labeling reagents (Applied Biosystems, Foster City, CA, USA) were added to the peptide samples, which were incubated at room temperature for 2 h.

The digested protein samples were separated using multidimensional liquid chromatography (LC). In the first dimension, the peptide mixtures were fractionated using an Ultimate LC system (Shimadzu 20AD, Kyoto, Japan) connected to an SCX column (Polysulfoethyl column, 2.1 mm × 100 mm, 5 u, 200 A; Nest Group, Southborough, MA, USA). A linear binary gradient from solvent A (10 mM KH_2_PO_4_ (Sinopharm Chemical Reagent Co. Ltd, Shanghai, China) and 25% acetonitrile (ACN, pH 2.6; Fisher Scientific, Fair lawn, NJ, USA), to solvent B (10 mM KH_2_PO_4_, 0.35 M KCl (Sinopharm Chemical Reagent Co. Ltd.), 25% ACN, pH 2.6 was applied: 0%–5% solvent B over 5 min, 5%–25% solvent B over 35 min, then 35%–100% solvent B over 10 min, with a flow rate of 200μl/min and detection at 214/280 nm. The entire run lasted 1 h, and 20 SCX fractions were collected. These fractions were vacuum dried (rotation vacuum concentrators, Christ RVC 2–25; Christ, Germany) and re-dissolved in 0.1% formic acid (Tedia, Fairfield, OH, USA) and 5% ACN.

Based on the SCX chromatograms, the 20 SCX fractions were combined into eight pools then desalted by ZORBAX 300SB-C18 column (5 μm, 300 Å, 0.1 × 150 mm; Microm, Miami, FL, USA). The pooled SCX fractions were automatically injected by a Famos autosampler and separated by an UltiMate capillary LC system (Dionex/LC Packings) and fractionated on a C18 PepMap main column (5μm, 300 Å, 0.1 × 150 mm; Microm, Miami, FL, USA) using a linear binary gradient (solvent A: 0.1% formic acid, 5% ACN; solvent B: 0.1% formic acid, 95% ACN). High Performance Liquid Chromatography (HPLC) linear gradients were from 0% solvent B (5 min) to 35% (70 min) and from 35% to 100% (120 min) at a flow rate of 0.3μl/min.

The peptides were eluted from the LC column and automatically deposited using a Probot spotting device. Mass spectrometry (MS) was conducted with a QSTAR XL instrument (Applied Biosystems).

### Data analysis

Peptide identification from the QSTAR XL data was carried out using the Paragon algorithm[18] in the ProteinPilot 4.2 software package (Applied Biosystems). MS/MS was performed on the four most abundant ions and the proteins identified by searching the SWISSPROT-vertebrate and National Center for Biotechnology Information (NCBI) databases. The following parameters were used for searching: trypsin as enzyme, fixed modification of methyl methanethiosulfate labeled cysteine, iTRAQ as sample type, no special factors, biological modification, thorough identification search, and fragmentation mass accuracy, which were built-in functions of ProteinPilot software, and the Paragon method was adopted. Then the name, function, International Protein Index (IPI), and similar characteristics were obtained from the Uniprot database. For protein-abundance ratios measured using iTRAQ, 1.5-fold up-regulation and 0.75-fold down-regulation change and the p-value < 0.05 (the p-value is generated from the peptide ratios used for quantitation) were set as the threshold for significant changes.

### Western blotting

The pools of proteins from each age group were mixed (4:1) with 5× sample buffer [0.5 ml 0.5 mM Tris.HCl (pH 6.8), 0.1 g SDS, 0.005 g bromophenol blue, 0.5 ml glycerol, 0.078 g DTT]. Proteins (40 μg) were boiled for 5 min and separated on SDS-PAGE in running buffer (25 mM Tris.HCl, pH 8.3, 1.4% glycine, 1 g SDS; Mini-PROTEAN Tetra Electrophoresis Cell, 0.75 mm thickness; Bio-Rad, Hercules, CA, USA) in two stages (30 min at 80 V, and 60 min at 120 V). The gels were transferred to Polyvinylidene Fluoride (PVDF) membranes (Millipore, Billerica, MA, USA) in ice-cold transfer buffer (25 mM Tris.HCl, pH 8.3, 1.4% glycine, 20% methanol; Mini Trans-Blot Electrophoretic Transfer Cell; Bio-Rad) at 200 mA for 1 h. Membranes were blocked with 5% non-fat milk (Becton, Dickinson and Company, Franklin Lakes, NJ, USA) in TBST (10 mM Tris.HCl, pH 8.0, 150 mM NaCl, and 0.1% Tween 20) for 90 min. Primary antibodies for Heat shock protein beta-1 (HSPB1) (diluted 1:400; Abcam, Cambridge, MA, USA), Apolipoprotein (APO)A1 (diluted 1:300; Biorbyt, Cambridge, UK), aldehyde dehydrogenase (ALDH)1A1 (diluted 1:300; Biorbyt), malate dehydrogenase (MDH)1 (diluted 1:300; Biorbyt), annexin (ANX)A6 (diluted 1:800; Sigma), and fast skeletal muscle troponin T isoform (TNNT3) (diluted 1:800; Sigma) were incubated overnight at 4°C. Membranes were washed twice for 10 min in TBST and once for 10 min in TBS. Horseradish peroxidase (HRP)-labeled anti-goat and anti-mouse secondary antibodies (Thermo Scientific Pierce, Waltham, MA, USA) were diluted 1:15,000 in TBST and incubated with the membranes for 90 min. After washing twice for 10 min in TBST and once for 10 min in TBS, immunoreactive proteins were visualized using a chemiluminescent HRP substrate (Millipore) in a dark room. The exposed films were analyzed for their gray-scale value using Image J.

## Results and Discussion

### Proteomic analysis of breast muscle

**Fig 1:**
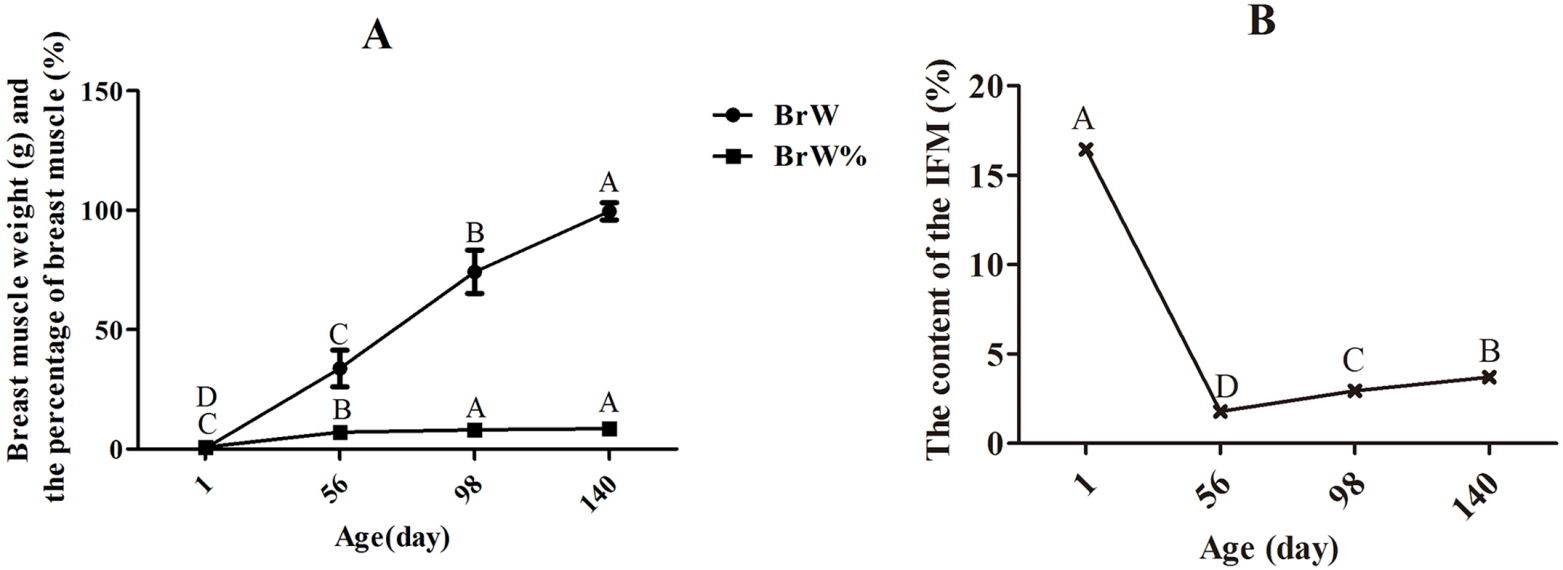
Changes in muscle growth and fat content in breast muscle at different ages. The data were analyzed by using one-way ANOVA and multiple comparison, and the results were shown as means ± SD. There are no bars on BrW % and IMF % because the SD was smaller than the symbols. (A) Changes in breast muscle growth with age. The weight and percentage of breast muscle (BrW, BrW%) increased significantly (P < 0.01) before day 98, but there was no difference in the percentage of breast muscle between days 98 and 140. (B) Content of IMF increased significantly (P < 0.01) after day 56, but was significantly higher at hatching than at other ages (P < 0.01).

Traits related to breast muscle weight and IMF content were measured (Fig 1A and 1B). The breast muscle absolute weight was obviously increased, but the breast muscle weight, relative to body weight, increased more slowly with age. Another study, using Beijing-You and western-type broilers, also showed that the breast muscle weights significantly increased with growth of the chickens[17]. Saneyasu, et al.[19]also investigated the change of body and breast muscle weights at 7, 14, 28, and 49 days of age, and showed significant increases in both with age. The relative weight of the breast muscle increased slowly, indicating a slight favoring of its growth over that of the whole body. Chartrin et al.[20] investigated lipid deposition in breast muscle of mule ducks at days 1 to 98 and found that there are two periods of IMF deposition. The first, from day 1 to 42, is when lipids (mainly phospholipids and cholesterol provided by the egg yolk) stored in the adipocytes during embryonic life were transferred to the muscle fibers and used for growth and energy requirements and the second, after day 42, is when muscle again stores lipids. The present result is consistent with that finding as the content of IMF was highest at day 1, decreased dramatically by day 56, then increased again from day 56 to 140.

The pooling strategy was adopted in this study, as it minimize the differences due to subject-to-subject variation and better identifies characteristics of the population[21]. Clustering showed that the protein expression profile was consistent for each of the repetitions (S1 Fig). Over 5000 proteins were identified and those accurately identified in breast muscle at days 1, 56, 98 and 140 were 494. Details of all accurately identified proteins as well as those at each sampled age are shown in S1 Table. Cluster analysis of all proteins expressed at different ages showed that the proteins in breast muscle at post-hatching ages (days 56, 98 and 140) were more similar than those at hatching, and the proteins at day 56 and day 98 were similar (S1 Fig).

**Fig 2:**
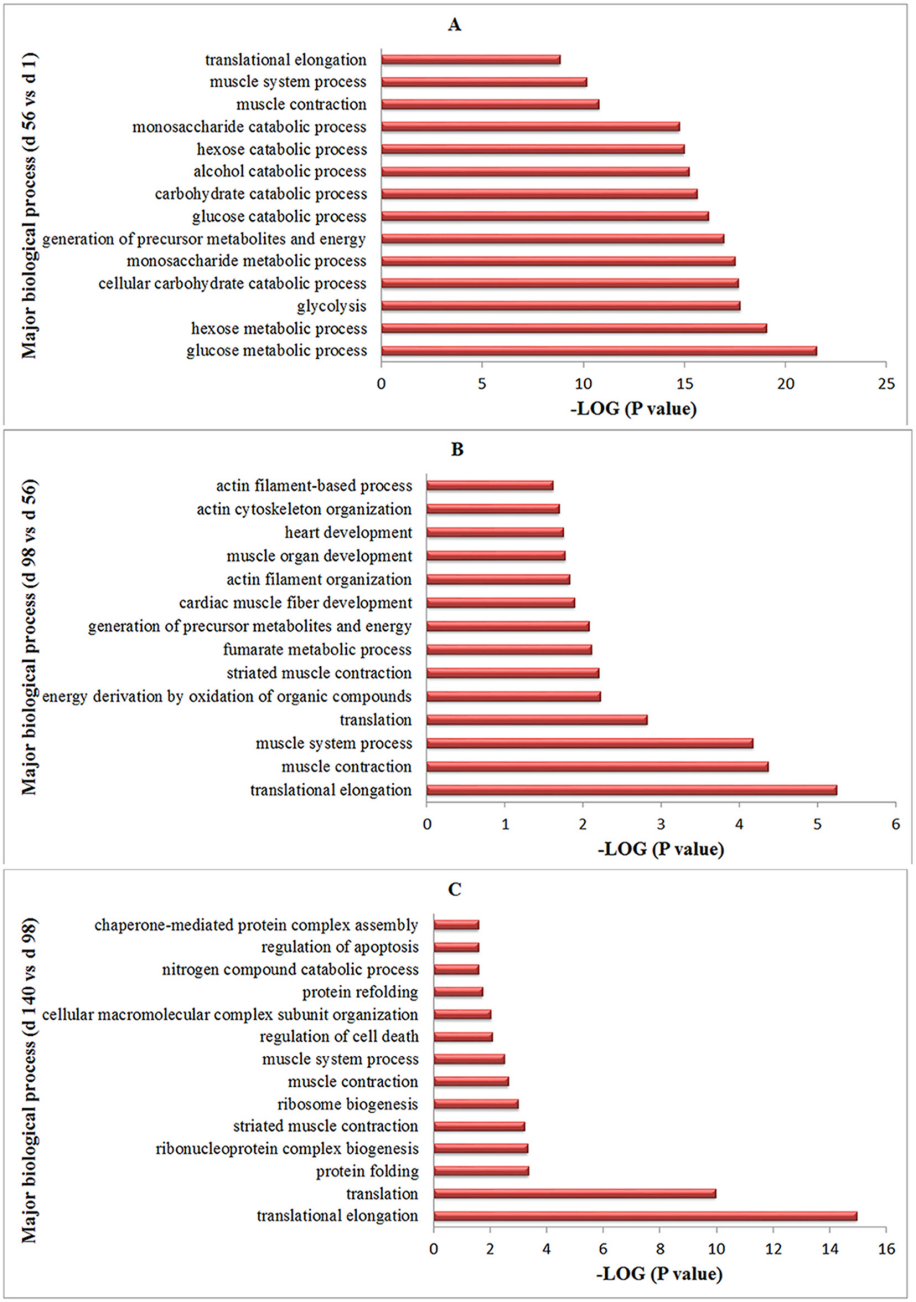
Biological processes of the differentially expressed proteins identified for three stages. The identified proteins were grouped into 15 categories according to their biological process for every stage (GO term with P-value < 0.05). Glucose and intermediary metabolism, muscle development, translation and protein folding were the major biological processes from day 1 to 56, day 56 to 98, and day 98 to 140.

Two hundred and four differentially expressed proteins were defined and analyzed (P < 0.05, indicating that the quantity in one pool was > 1.5 or < 0.7, compared to the other pool, for each pairwise comparison) (S2 Table). To gain insight into the changes between each stage, four groups of proteins by Gene Ontology analysis were compared: day 56 vs. day 1, day 98 vs. day 56, and day 140 vs. day 98 (S3 Table and Fig 2). Proteins related to glucose and intermediary metabolism were abundant from day 1 to 56; proteins involved in muscle development were abundant from day 56 to 98; and from day 98 to 140, translation and protein folding processes were abundant. The protein expression profiles were similar at the three post-hatching ages (days 56, 98 and 140) but differed from those at day 1 by clustering analysis. The molecular mechanisms of muscle development and IMF deposition are different at hatching and post-hatching stages.

### Highly upregulated or downregulated proteins in hatchlings compared to post-hatching chickens

The proteins were defined as age-specific, highly upregulated or downregulated when the content differed >10 fold compared to that at other ages. As shown in S4 Table, many proteins were highly upregulated at day 1, such as ADP-ribosylhydrolase like 1 (ADPRHL1), alpha-2-HS-glycoprotein (AHSG), apolipoprotein A (APOA1, APOAIV), histone family (H1, H2B-VII, HIST2H2AC, H4), thymocyte nuclear protein 1 (THYN1), myosin light polypeptide 6 (MYL6), isocitrate dehydrogenase [NADP](IDH), peptidyl-prolyl cis-trans isomerase (PPIA), sarcalumenin (SRL), tubulin beta-7 chain (TUB7) *inter alia*. Some proteins were highly downregulated at day 1 (S4 Table), which were mostly involved in energy metabolism and muscle development; for example, adenylate kinase isoenzyme 1 (AK1), fructose-bisphosphate (ALDOA.ALDOB.ALDOC), creatine kinase S-type, mitochondrial (CKMT2), desmin (DES), fructose-1, 6-bisphosphatase 2 (FBP2), glyceraldehyde-3-phosphate dehydrogenase (GAPDH), glycogen phosphorylase (GPH1), glucose-6-phosphate isomerase (GPI), L-lactate dehydrogenase A chain (LDHA), phosphoglycerate kinase 1 (PGK1), malate dehydrogenase 1and 2 (MDH1, MDH2), malic enzyme 1 (ME1), phosphoglycerate mutase 1(PGAM1), phosphorylase B and L chain (PYGB,PYGL), acylphosphatase (ACYP), alpha-actinin-2 (ACTN2), troponin C, skeletal muscle (TNNC2), troponin I, fast skeletal muscle (TNNI2), fast skeletal muscle troponin T isoform (TNNT3), triosephosphate isomerase 1 (TPI1), tropomyosin alpha-1 chain (TPM1) etc.

### Over-represented pathways in breast muscle from day 1 to 56

Between days 1 and 56, breast muscle weight increased about 100-fold and IMF decreased about 100-fold. Thus, this is a critical stage for muscle growth and depletion of IMF (Fig 1A and 1B).

**Fig 3:**
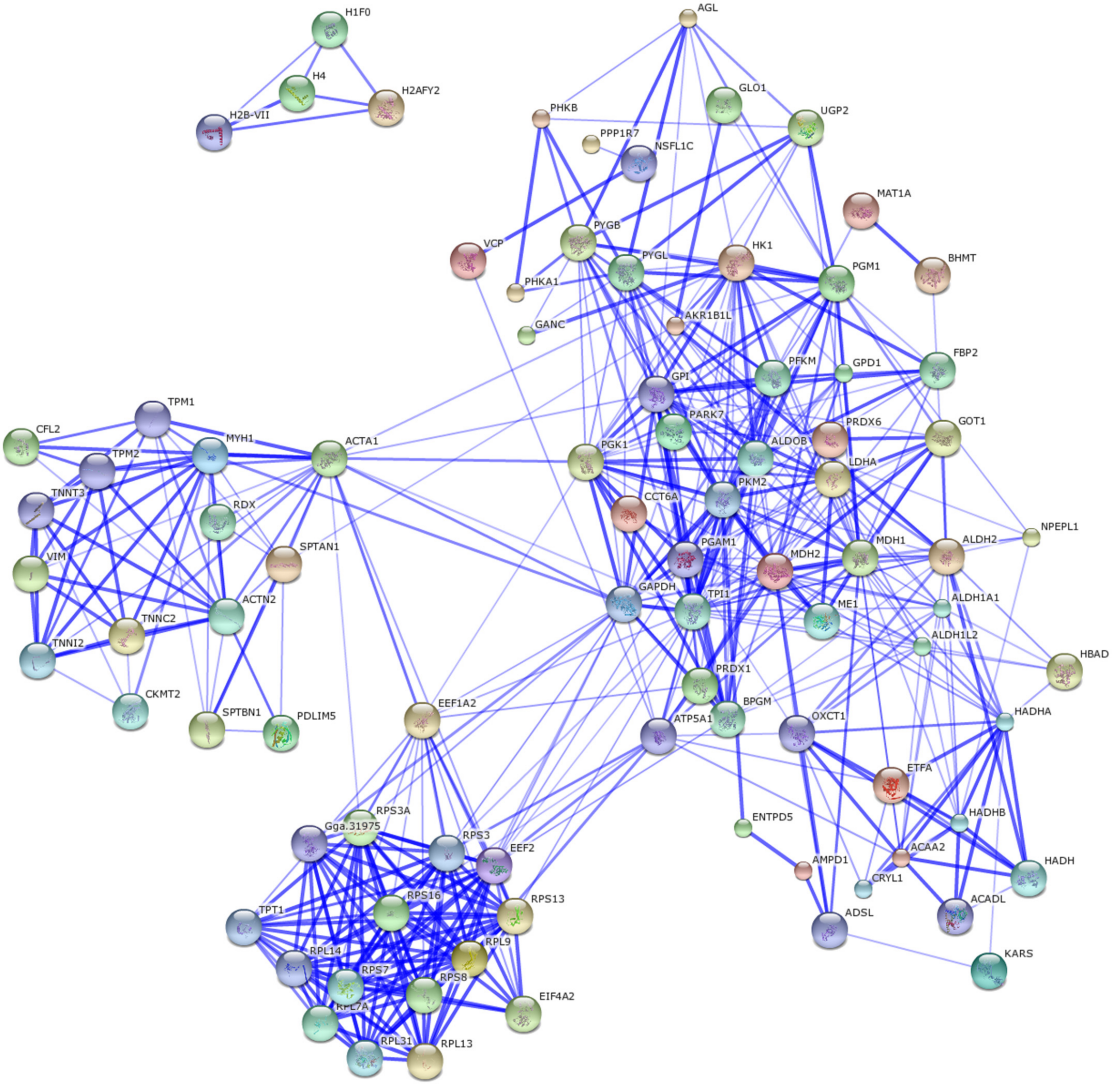
Protein interaction network of differentially expressed proteins between day 1 and 56. Two functional modules were apparent in the network, forming tightly connected clusters. The first functional module included the proteins related to metabolic pathways, and the second included the proteins involved in the ribosomes.

Comparing the protein profiles at days 1 and 56, 191 differing proteins were identified (the fold difference was > 1.5 or < 0.7). The 19 metabolic pathways were enriched during this fast growing stage based on a KEGG pathway analysis (S5 Table). The protein–protein interaction network of the differentially expressed proteins identified for this interval (days 56 vs. 1) was also analyzed by web-tool STRING 10.0 (http://string-db.org); there were two functional modules (Fig 3). The first related to metabolic pathways, including glycolysis/gluconeogenesis pathway, insulin signaling pathway, and lipid metabolic pathway. This module might relate to the significant changes in breast muscle, such as fast muscle growth and IMF deposition (Fig 1A and 1B). The second module involved the ribosomes.

### Active pathways and key differentially expressed proteins at day 1

Within the 19 metabolic pathways, some differentially expressed proteins that were highly expressed at day 1 compared to day 56 were involved in the lipid metabolic pathway, such as valine, leucine and isoleucine degradation, and fatty acid degradation and elongation in mitochondria.

Of eight differentially expressed proteins involved in the fatty acid degradation pathway, five (CPT-2, ACADL, HADHA, HADHB and ACAA2) were more abundant at day 1 than at day 56, and ALDH1A1 and ALDH2 were significantly lower at day 1 compared to day 56 (Abbreviations used in this paper are listed in Tables 1 and 2). It was deduced that lipid oxidation was more active and fatty acid synthesis was less active at day 1 compared to day 56.

**Table 1:** Abbreviations used.

Abbreviations	Gene name	Protein name
ACAA2	acetyl-CoA acyltransferase 2	uncharacterized protein
ACADL	acyl-CoA dehydrogenase, long chain	uncharacterized protein
ACTA1	actin, alpha 1	actin, alpha
ACTN4	actinin, alpha 4	alpha-actinin-4
ALDH1A1	aldehyde dehydrogenase 1 family, member A1	retinal dehydrogenase 1
ALDH2	aldehyde dehydrogenase 2 family	uncharacterized protein
ANXA2	Annexin A2	annexin A2
APOA1	apolipoprotein A-I	apolipoprotein A-I
APOAIV	apolipoprotein A-IV	apolipoprotein AIV
CPT-2	carnitine O-palmitoyltransferase 2	uncharacterized protein
DES	desmin	desmin
HADH	hydroxyacyl-CoA dehydrogenase	uncharacterized protein
HADHA	hydroxyacyl-CoA dehydrogenase/3-ketoacyl-CoA thiolase/enoyl-CoA hydratase (trifunctional protein), alpha subunit	uncharacterized protein
HADHB	hydroxyacyl-CoA dehydrogenase/3-ketoacyl-CoA thiolase/enoyl-CoA hydratase (trifunctional protein), beta subunit	uncharacterized protein
KBTBD10	kelch repeat and BTB (POZ) domain containing 10	uncharacterized protein
LAMA2	laminin, alpha 2	uncharacterized protein
LAMB1	laminin, beta 1	laminin subunit beta-1
LMNA	lamin-L(III)-like	lamin-A
MYBPC1	myosin binding protein C	uncharacterized protein
MYBPC2	myosin binding protein C	myosin-binding protein C, fast-type
MYBPH	myosin binding protein H	uncharacterized protein

**Table 2:** Abbreviations used.

Abbreviations	Gene name	Protein name
MYH6	myosin, heavy chain 6	myosin heavy chain
MYOM2	myomesin 2	uncharacterized protein
MYLPF	myosin light chain, phosphorylatable	myosin regulatory light chain 2
MYOZ1	myozenin 1	uncharacterized protein
MYOZ3	myozenin 3	uncharacterized protein
PDLIM3	PDZ and LIM domain 3	PDZ and LIM domain protein 3
SPTAN1	spectrin, alpha, non-erythrocytic 1	spectrin alpha chain
TNNC2	troponin C type 2	troponin C
TNNI2	troponin I type 2	troponin I
TNNT3	troponin T type 3	troponin T
TPM1	tropomyosin alpha-1 chain	tropomyosin alpha-1 chain
TPM2	tropomyosin beta chain	tropomyosin beta chain
TTN	myopalladin	connectin
TUB7	tubulin beta-7 chain	tubulin beta-7 chain

The differentially expressed proteins, APOA1 and APOAIV, were identified (S4 Table), which was consistent with previous studies[5]. The reason may be that a large number of lipoproteins take up cholesterol from the yolk sac membrane at hatching[5,22,23], and the production of the APOs was stimulated by lipoproteins. Synthesis of APOAI in the skeletal muscle of hatchling chicks acts as a local lipid transporter in early post-hatching development[24]. This is consistent with the phenotype where the content of IMF at day 1 is about 8 times higher than that at day 56 (Fig 1B).

Expression of histone family and ribosomal family proteins (S6 Table) differed greatly between hatching and post-hatching (56, 98 and 140 days). Histones play a pivotal role in regulating gene expression by controlling the access of key regulatory factors and complexes to chromatin, which is essential for transcription, DNA replication, DNA repair and DNA recombination [25–29]. The organization of chromatin is considered to be regulated by post-translational modification of histones, such as methylation, acetylation, phosphorylation and ubiquitination[28]. Many studies have shown that myogenesis is controlled through sequential chromatin regulation by the selection of the histone variant and the appropriate histone modification [30–33]. For example, in mouse embryos, a bivalent modification of H3K4me3 and H3K27me3 was formed on H3.3-incorporated skeletal muscle genes before embryonic skeletal muscle differentiation [34]. Ribosomal proteins, in conjunction with rRNA, make up the ribosomal subunits involved in the cellular process of translation and protein biosynthesis. It has been demonstrated that differential mRNA translation controls protein expression of specific subsets of genes during myogenesis, and one of a subset of transcripts that is enriched for mRNAs encoding ribosomal proteins is regulated at the translational level [35]. Ribosomal proteins were also highly expressed during mature adipogenesis[36]. A large group of ribosomal proteins was identified in chickens (S6 Table), which may partly explain why differentiation of myocytes and preadipocytes within muscle occurs mainly before hatching.

### Active pathways and key differentially expressed proteins at day 56

Many metabolism-related proteins were more abundant at day 56 than at day 1. There were 24 upregulated proteins, related to cytoskeleton and actin binding, including actin, cofilin, desmin, actinin, myosin, calpain, calmodulin, troponin, myomesin and myozenin.

These proteins were involved in muscle-development-related pathways, such as glycolysis/gluconeogenesis, hypertrophic cardiomyopathy (HCM), insulin signaling, cardiac muscle contraction and dilated cardiomyopathy (DCM). Sixteen upregulated proteins related to the glycolysis/gluconeogenesis pathway were more abundant at day 56, and eight proteins in the HCM pathway and six (LMNA, DES, TTN, TPM1, TPM2, MYH6) were highly abundant at day 56.

The period from day 1 to 56 is a fast growing stage for skeletal muscle (Fig 1A). The identified proteins play a critical role in all skeletal and cardiac muscle in the early stages of development[37,38]. Specifically, the energy from glycolysis/gluconeogenesis metabolism is needed for developing skeletal muscle. Insulin signaling pathway proteins are involved in the proliferation and differentiation of preadipocytes and myocytes[39–41], and are prominent for coordinating myofiber growth, muscle hypertrophy and muscle regeneration[42–45]. calmodulin (CALM), connectin/titin (TTN) and troponin C (TnC) are activated by the second messenger Ca^2+^ and stimulate expression of troponin I (TnI), troponin T (TnT), actin, myosin and muscle development[46–50]. In the present study, the amount of TTN, TnC (TNNC2), TnI (TNNI2), TnT (TNNT3), actin (ACTA1), myosin (MYBPC1, MYBPC2, MYBPH, MYLPF, MYH6), M line (MYOM2) and Z line (MYOZ1, MYOZ3) was enhanced, related to muscle development at day 56. Muscle contraction and hypertrophy are the main mechanisms influencing muscle development, along with the insulin signaling and HCM pathways at day 56.

### Over-represented pathways in breast muscle from day 56 to 98 (market age)

From day 56 to 98, breast muscle weight and IMF about doubled and thus fast growth of muscle and IMF deposition continues (Fig 1A and 1B). For the local slow-growing chickens used here, market age is around day 98.

**Fig 4:**
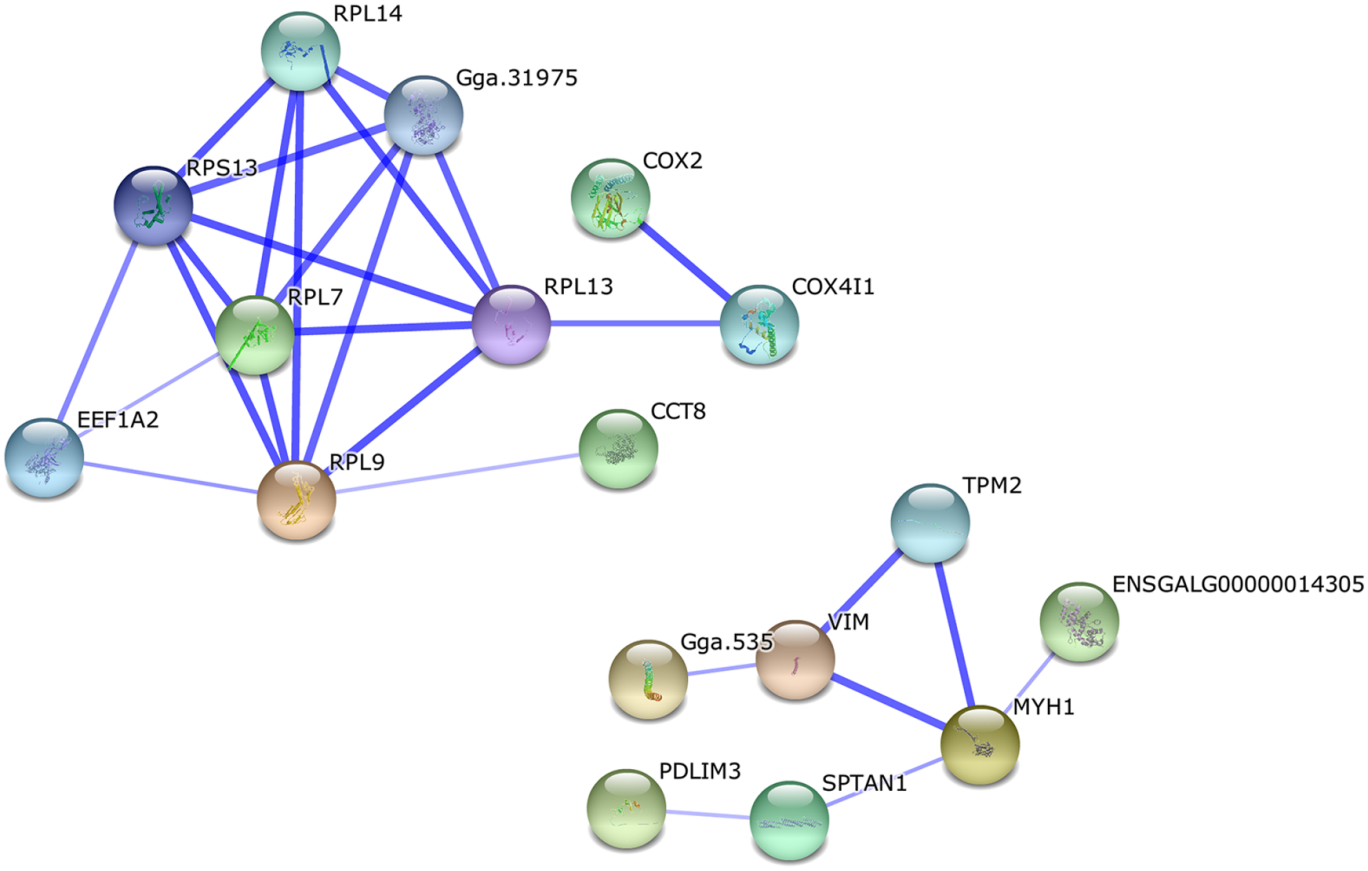
Protein interaction network of differentially expressed proteins between days 56 and 98. There was only one significant functional module in the network, which was related to the ribosomes.

There were 44 differentially expressed proteins identified when comparing days 56 and 98. Seven significant enrichment pathways were identified by KEGG analysis (S5 Table), of which six are involved in muscle development. The only significant functional module in the network analysis of protein interaction (Fig 4), however, related to the ribosomes.

There were 12 differentially expressed proteins related to cytoskeleton and actin binding, which was consistent with the high muscle growth rate at this stage. Four more abundant proteins at day 98 than at day 56 related to HCM and three related to focal adhesion. This suggests that the muscle development at this stage was closely connected with muscle growth, and these proteins may play important roles in focal adhesion and HCM during skeletal muscle development. The proteins included ACTN4, extracellular matrix proteins (LAMA2 and LAMB1), MYH6, TTN, DES and TPM2. Previous studies have made inroads into understanding the mechanism underlying muscle development. The enzymes related to cytoskeletal protein binding, including MYBPC2, MYBPC1, PDLIM3, ANXA2, SPTAN1, KBTBD10 and TUB7 were highly abundant in breast muscle at day 98 compared to day 56. ANXA2 is a Ca^2+^-binding protein implicated in several biochemical processes, including cell proliferation, ion-channel activation, cytoskeleton rearrangement, cell–cell interactions and the bridging of membranes [51–54]. ANXA2 forms junctions between lipid bilayer structures through molecular bridging of their external leaflets[55,56]. From day 56 to 98, focal adhesion, tight junctions and HCM play an important role in muscle development. Extracellular matrix proteins had a key role in muscle growth at day 98, which was different from day 56. ANXA2 may play an important role in lipid metabolism.

### Over-represented pathways in breast muscle from day 98 to 140

From day 98 to 140, breast muscle weight and IMF increased about 1.3-fold, so muscle growth and IMF deposition are slowing from that occurring in the previous phase (Fig 1A and 1B). For the local slow-growing chickens used here, day 140 is near sexual maturity.

**Fig 5:**
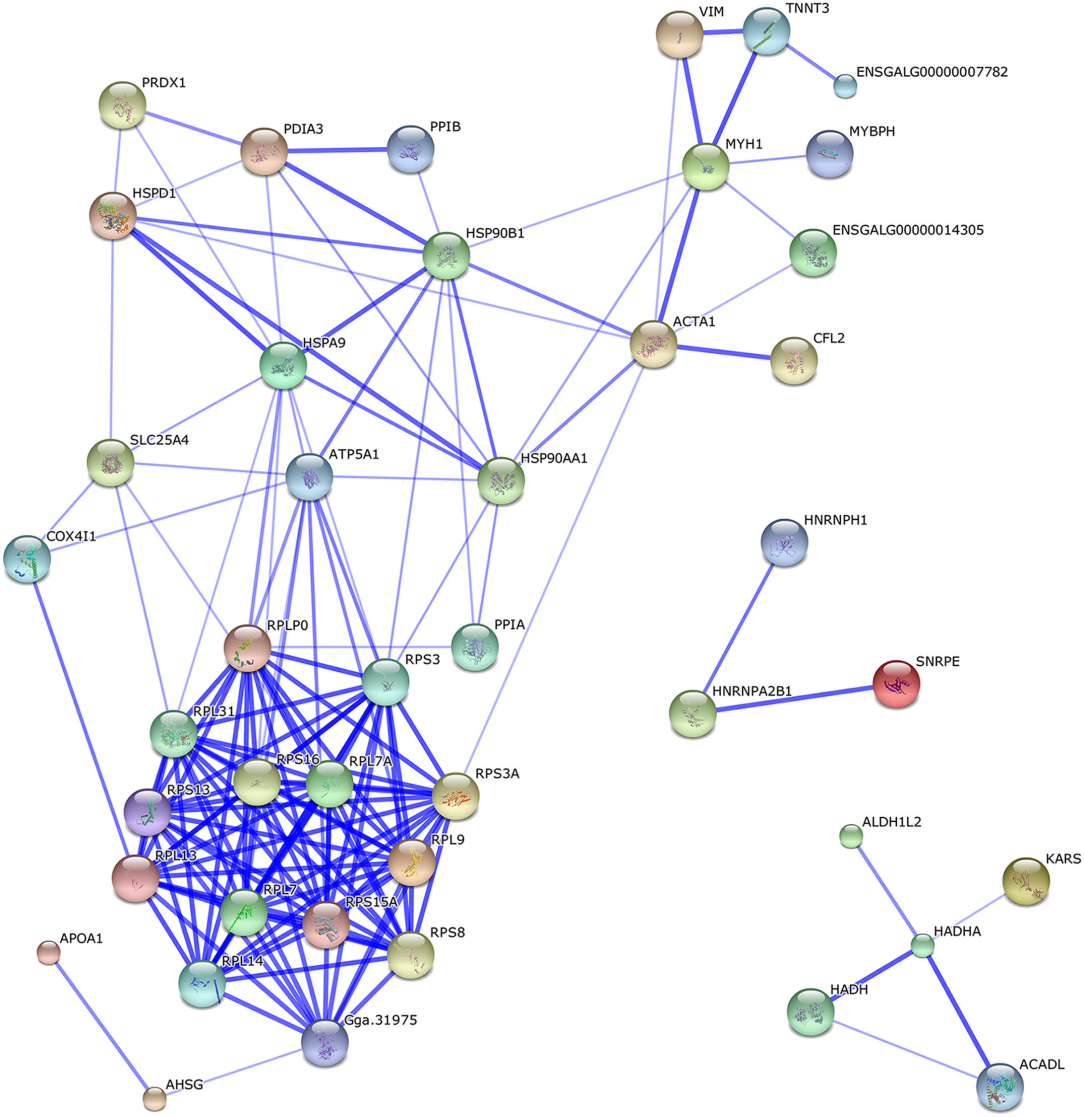
Protein interaction network of differentially expressed proteins between days 98 and 140. There was only one significant functional module in the network, which involved ribosomes.

Comparing the proteins at days 98 and 140, 58 were identified as being differentially expressed with cytoskeletal and ribosomal proteins being less abundant at day 140 than at day 98. The cytoskeleton is present in all cells, and plays important roles in cellular processes such as differentiation and apoptosis. As key regulators of cellular architecture, cytoskeletal components contribute to physical processes such as adhesion and migration[57–59]. Ribosomal proteins relate to the cellular processes of translation and protein biosynthesis. This decrease in abundance suggests that the rate of growth and the metabolic processes were slower at 140 than at 98 days. The only functional module clustering in the protein–protein interaction network involved the ribosomes (Fig 5).

Three pathways were identified by KEGG analysis, with 58 differentially expressed proteins, including those of fatty acid metabolism and the proteins related to lipid metabolism, ACADL, APOA1 and HADH, were more abundant at day 140 compared to day 98 (S5 Table). So, the capacity for lipid production was increased and that of oxidation decreased, resulting in lipid accumulation, perhaps explaining the higher IMF content in mature birds compared to younger birds.

### Verification of content of key proteins by Western blotting

**Fig 6:**
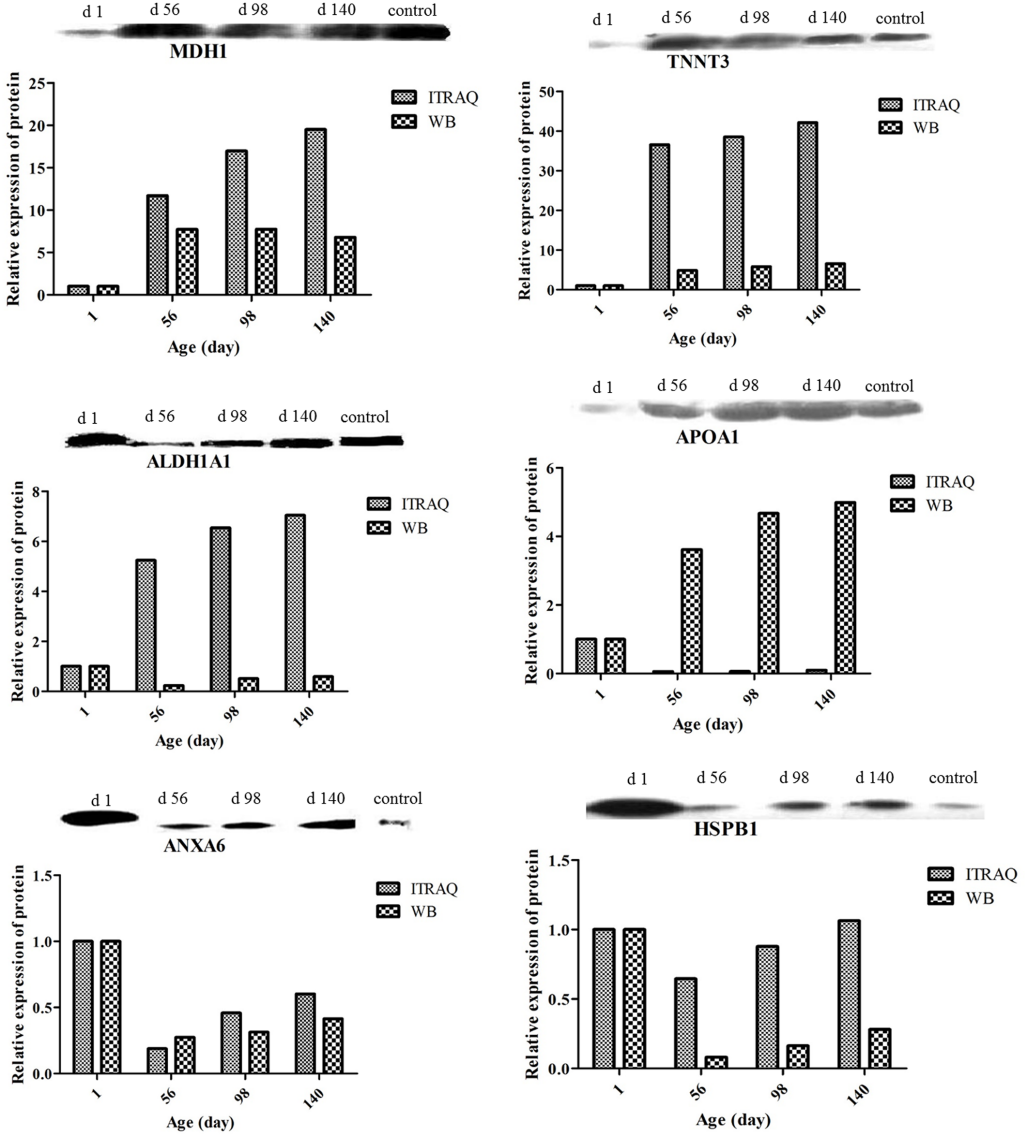
Verification of content of key proteins by Western blotting. Comparison between levels of MDH1, TNNT3, ALDH1A1, ANXA6, APOA1 and HSPB1 determined by Western blotting and iTRAQ. Values, within methods, were normalized to those in hatchling chickens.

To validate the results of the iTRAQ testing, Western blotting was used to examine the relative contents of six key functional proteins at the different ages (Fig 6). Two proteins (MDH1 and TNNT3) related to muscle development, and four proteins (ALDH1A1, ANXA6, APOA1, HSPB1) related to lipid metabolism. There was acceptable consistency between the results of Western blotting and the fold-change of differentially expressed proteins from iTRAQ analysis. No internal reference was used here in the Western blotting because the abundance of β-actin, β-tubulin, histone and GAPDH all differed significantly at hatching and the post-hatching ages. The control sample used for Western blotting was a composite of breast muscle proteins made by pooling the different ages. MDH1 plays an important role in transporting NADH equivalents across the mitochondrial membrane, controlling tricarboxylic acid (TCA) cycle pool size and providing contractile function[60], so the increased expression of MDHs is required for the high demands of energy metabolism in developing tissues[61], especially in those with high metabolic rate such as heart, skeletal muscle, and brain. Troponin-mediated Ca^2+^-regulation governs the actin-activated myosin motor function which plays a key role in the regulation of striated muscle contraction in vertebrates[62]. Point mutations in the *cTnT* gene have been found in human familial hypertrophic cardiomyopathy[63], and the expression of TnT isoform is regulated during heart and muscle development and adaptation[64], suggesting that TnT plays an important role in muscle growth and function. ALDHs are known to participate in oxidizing a plethora of endogenous and exogenous aldehydes[65]. ALDH1A1 was up-regulated in omental and intramuscular preadipocytes during differentiation[66], and the increased levels of ALDH1A1 in the obese omental fat might be involved in fat accumulation[67]. Annexin A6 (AnxA6) is a Ca^2+^ and phospholipid binding protein that acts as a scaffolding protein and regulates cholesterol transport along endo- and exocytic pathways. Loss of AnxA6 alters both lipid and glucose homeostasis, resulting in increased lipolysis and high density lipoprotein increased in *AnxA6* KO mice[68]. Apolipoprotein A1 (apoA1) is the major apolipoprotein constituent of the high-density lipoprotein (HDL) and is involved in reverse cholesterol transport[69]. Variants in the apolipoprotein A1 (*APOA1*) gene play an important role in the regulation of lipid transport[70–73]. Synthesis of APOAI in the skeletal muscle of hatchling chicks acts as a local lipid transporter for early post-hatching development[24]. Heat shock protein beta 1 (HSPB1), a member of the heat-shock family of proteins, is a relatively small (27 kDa) molecular chaperone protein associated with cellular development, differentiation, and signal transduction[74]. HspB1 and its regulator genes (*FAS*, and *AGT*) were shown to be good candidate genes associated with intramuscular fat content in the longissimus muscle of Korean cattle[75].

## Conclusion

In summary, the present study provides a useful resource for further investigating the roles of proteins expressed differentially in skeletal muscle at different developmental stages. Such efforts will enable better understanding of the molecular mechanisms of muscle development in chickens. The changes in protein abundance with age have not been documented previously, and the extent of the changes found here was unexpected. This study is the first step in understanding post-hatching development on a proteome-wide scale, and indicates the complexity of such an analysis. In addition, the present results suggest that APOA1 and HSPB1 may be useful as molecular markers of IMF deposition in chickens.

## Supporting Information

S1 Fig The hierarchical clustering results of all proteins at different stages. (TIF)

S1 Table Details of all accurately identified proteins in breast muscle at all different stages. (XLSX)

S2 Table Two hundred and four significantly different proteins. (XLSX)

S3 Table Differentially expressed proteins between each stage. (XLSX)

S4 Table Highly upregulated or downregulated proteins in hatchling compared to post-hatching chickens. (XLSX)

S5 Table KEGG pathway enriched at different stages. (XLSX)

S6 Table Some protein family specs63ific or highly expressed at d 1. (XLSX)
