# Supplementary figures and images for: Protein Profiles for Muscle Development and Intramuscular Fat Accumulation at Different Post-Hatching Ages in Chickens
Source: PLoS One. 2016 Aug 10;11(8):e0159722. doi: 10.1371/journal.pone.0159722 (PMC4980056; doi:10.1371/journal.pone.0159722)

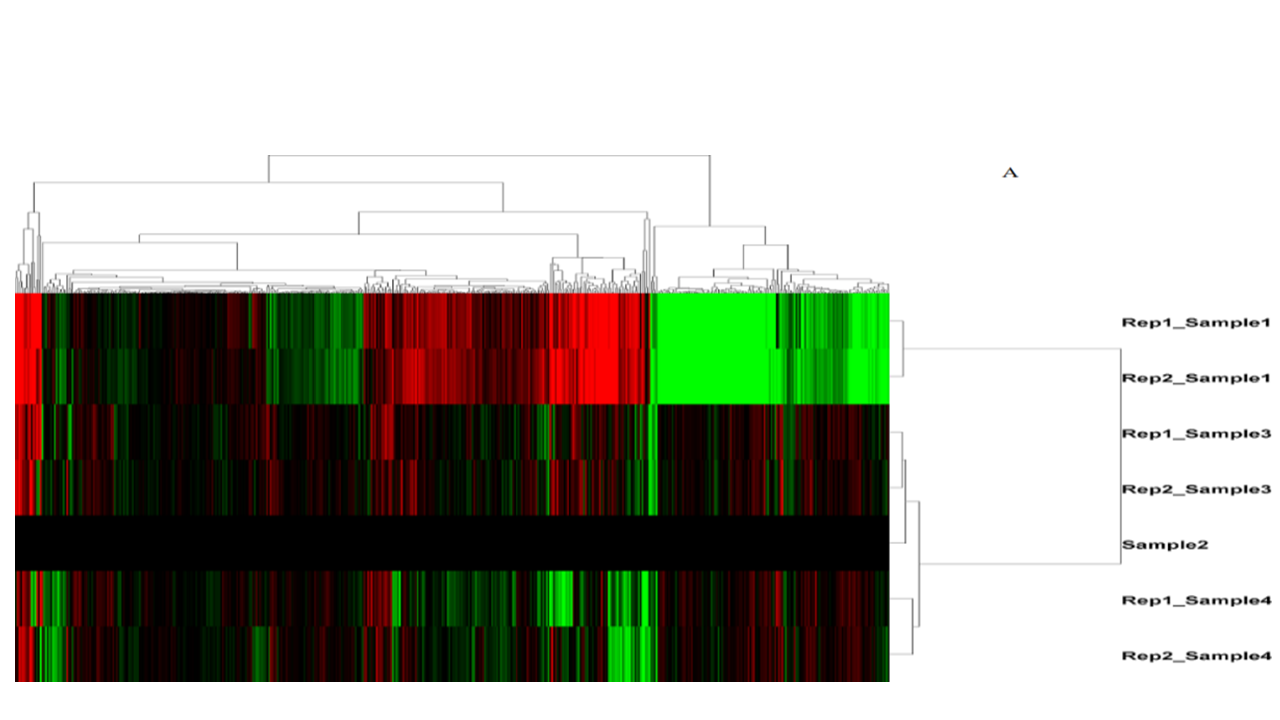

Supplement: S1 Fig — (TIF) [file pone.0159722.s001.tif]
